# Supplementary material for: High Prevalence and Clinical Associations of Vitamin D Deficiency in Inflammatory Bowel Disease: Evidence from a Tertiary Center Cohort
Source: Nutrients. 2025 Nov 25;17(23):3698. doi: 10.3390/nu17233698 (PMC12694141; doi:10.3390/nu17233698)
Supplement: Supplementary file 1 [file nutrients-17-03698-s001.zip › nutrients-3985295-supplementary.pdf]

Supplementary Material

Supplementary Table S1. Comparison of clinical and laboratory characteristics between patients with and without vitamin D deficiency

Variable	Vitamin D deficiency N=67			Without Vitamin D deficiency N=117			
	**N**	**%**	**C.I.**	**N**	**%**	**C.I.**	**p-value**
**Gender** (Male)	34	50.7	0.382-0.632	63	54.3	0.448-0.636	0.642
**Smoking** Yes/No/Prior	19/37/7	29.2%/60.0%/10.8%	0.186-0.418/0.471-0.720/0.044-0.209	43/58/15	37.1%/50.0%/12.9%	0.283-0.465/0.406-0.594/0.074-0.204	0.431
**IBD family history** Yes/No	1/65	1.5%/98.5%	0.000-0.082	8/107	7.0%/93.0%	0.031-0.132	0.105
**IBD Type** CD/UC/IC	39/27/1	58.2%/40.3%/1.5%	0.455-0.702/0.285-0.530/0.000-0.080	54/57/6	46.2%/48.2%/5.1%	0.369-0.556/0.394-0.581/0.019-0.108	0.116/0.270/0.196
**IBD Hospitalization** Yes/No	29/31	48.3%/51.7%	0.352-0.616	46/54	46.0%/54.0%	0.360-0.563	0.775
**IBD surgery** Yes/No	14/46	23.3%/76.7%	0.134-0.360	15/85	15.0%/85.0%	0.086-0.235	0.185
**Without IBD treatment**	2	3%	0.004-0.104	5	4.5%	0.015-0.101	0.621
**Amino salicylates**	21	31.3%	0.206-0.438	46	41.1%	0.319-0.508	0.193
**Immunomodulators**	5	7.5%	0.025-0.166	11	9.8%	0.050-0.169	0.592
**Corticosteroids**	7	10.4%	0.043-0.203	9	8%	0.037-0.147	0.584
**Biologic Agents**	60	89.6%	0.797-0.957	82	73.2%	0.640-0.811	**0.009**
	**Median**	**IQR**	**C.I.**	**Median**	**IQR**	**C.I.**	**p-value**
**Age** (years)	43.00	33.00-60.00	0.470-0.715	46.50	35.25-62.00	0.517-0.701	0.259
**Age at diagnosis** (years)	34.00	23.00-46.00	0.577-0.807	32.00	22.25-45.00	0.670-0.833	0.719
**BMI**	23.60	20.14-27.50	0.622-0.859	24.90	22.20-27.77	0.881-0.983	0.145

**Hb** (g/dL)	12.90	11.50-14.40	0.397-0.646	13.80	12.88-14.62	0.953-1.000	**0.005**
**WBC** (10^9/L)	7380	5970-9290	0.674-0.881	6540	5600-8640	0.913-0.990	0.146
**PLT** (10^9 /L)	306000	233000-381000	0.815-0.966	264000	227000-332250	0.665-0.830	**0.005**
**CRP** (mg/L)	0.60	0.20-1.65	0.854-0.983	0.32	0.20-1.02	0.950-1.000	0.075
**ESR** (mm/h)	20	12-33	0.773-0.965	12	9-24	0.885-0.991	**0.014**
**SGOT** (U/L)	18	15-21	0.778-0.947	20	16-25	0.924-0.994	**0.009**
**SGPT** (U/L)	17	12-26	0.760-0.937	21	14-30	0.952-1.000	0.121
**γ-GT** (U/L)	16.0	12.0-27.5	0.918-1.000	16.0	13.0-26.0	0.923-0.994	0.809
**Albumin** (g/dL)	4.2	3.8-4.4	0.920-1.000	4.3	4.1-4.5	0.002-0.066	**0.011**
**Fecal Calprotectin** (μg/g)	272	120-654	0.349-0.968	75	36-332	0.587-0.998	0.215
**Mayo score**	2	0-6	0.554-0.843	1	1-5	0.662-0.862	0.076
**CDAI score**	58	25-111	0.627-0.904	64	37-103	0.485-0.751	0.880
**HBI score**	2.50	1.75-4.25	0.847-0.999	2.00	1.00-3.00	0.821-0.979	0.197
**Numbers of biologic agents**	1	1-2	0.593-0.820	1	0-1	0.853-0.963	0.070

Abbreviations: *N, number; C.I., Confidence Interval;* x^2*, chi-square test; IBD, inflammatory bowel disease; CD, Crohn's disease; UC, Ulcerative Colitis; IC, indeterminate colitis; IQR, interquartile range; BMI, body mass index; Hb, hemoglobin; WBC, White Blood Cell; PLT, platelets; CRP, C-reactive protein; ESR, erythrocyte sedimentation rate; SGOT, aspartate aminotransferase; SGPT, alanine aminotransferase; γ-GT, gamma-glutamyl transferase; CDAI, Clinical Disease Activity Index; HBI, Harvey-Bradshaw Index*.
